# Spike pattern recognition by supervised classification in low dimensional embedding space

**DOI:** 10.1007/s40708-016-0044-4

**Published:** 2016-03-16

**Authors:** Evangelia I. Zacharaki, Iosif Mporas, Kyriakos Garganis, Vasileios Megalooikonomou

**Affiliations:** 1 Department of Computer Engineering and Informatics, University of Patras, Patras, Greece; 2 Center for Visual Computing, CentraleSupélec/Galen Team, INRIA, Paris, France; 3 St. Luke’s Hospital, Thessaloniki, Greece

**Keywords:** Spike detection, Epilepsy, Pattern recognition, Manifold learning, Dimensionality reduction

## Abstract

Epileptiform discharges in interictal electroencephalography (EEG) form the mainstay of epilepsy diagnosis and localization of seizure onset. Visual analysis is rater-dependent and time consuming, especially for long-term recordings, while computerized methods can provide efficiency in reviewing long EEG recordings. This paper presents a machine learning approach for automated detection of epileptiform discharges (spikes). The proposed method first detects spike patterns by calculating similarity to a coarse shape model of a spike waveform and then refines the results by identifying subtle differences between actual spikes and false detections. Pattern classification is performed using support vector machines in a low dimensional space on which the original waveforms are embedded by locality preserving projections. The automatic detection results are compared to experts’ manual annotations (101 spikes) on a whole-night sleep EEG recording. The high sensitivity (97 %) and the low false positive rate (0.1 min^−1^), calculated by intra-patient cross-validation, highlight the potential of the method for automated interictal EEG assessment.

## Introduction

The detection of epileptiform discharges in interictal EEG is important for the diagnosis of epilepsy. Interictal spikes are brief (<250 ms), morphologically defined events observed in the EEGs of patients predisposed to spontaneous seizures of focal onset [1]. The spikes are generated by the synchronous discharges of a group of neurons in a region referred to as the epileptic focus [1]. The detection of spikes is difficult to accomplish due to their similarity to waves that are part of normal EEG or artifacts and the wide variability in spike morphology and background between patients [2]. Also the spike definitions are imprecise and vary among neurophysiologists who often do not mark the same events as spikes. A comprehensive review on automated spike detection methods is presented in [3], and later updated in [4], while a comparative analysis is presented by Wilson and Emerson [5], and Halford [6]. According to the review studies, the methods are classified into different categories based on the spike detection criterion, while many approaches use a combination of methods in a multi-stage framework. In more details, some methods extract distinctive attributes of the spikes, such as height and duration, mimicking the criteria used by the neurophysiologists [7] or utilize knowledge-based rules (spatial and temporal) [8, 9]. Other methods characterize the spikes in time or frequency domain and through morphological analysis decompose the EEG signal [10] or assume local stationarity of the noise and detect spikes as deviation from that stationarity by applying parametric models [11, 12]. There are methods in which a template (created by averaging expert-defined spikes) is used for matching against the extracted EEG waveforms [12]. Other studies use independent component analysis [13], apply artificial neural networks (ANNs) [3, 14], clustering techniques [15], or classification methods [16].

Despite the plethora of methods, spike assessment is often still performed visually due to increased false discovery rate of most methods. Among the methods with highest sensitivity (>0.92) and selectivity (>0.8) are the ones reported in [14, 17–20]. However, their accuracy is not easily comparable. Some methods have not been evaluated on long-term recordings but on preselected (usually by neurologists with experience in EEG reading) EEG segments of epileptiform and non-epileptiform discharges [17, 20]. Since these EEG segments have distinguishable (visually identifiable) patterns, it is expected to obtain higher accuracy than long EEG recordings, which possibly include artifacts and/or unclear EEG patterns. In [14], Gabor and Seyal applied ANNs for the identification of epileptiform transients in EEG signals. Their method is not completely automated since, prior to selection of training patterns, a user has to identify the peak of a spike or sharp wave that will be used for training, as well as the duration of the rising phase and the falling phase. Selection of training patterns was accomplished by the user after viewing a graphic display of the EEG signal. ANNs were also used in [19], after a template matching method where the user visually selects a few spikes from a set of test signals. Features of the signal were obtained by wavelet transformation and subsequently were used to train a feed-forward ANN. Context information of adjacent channels was utilized to reject artifacts. In [18], an expert system is proposed which exploits multi-channel EEG, as well as electrocardiogram (ECG), electrooculogram (EOG), and electromyogram (EMG) channels. The use of multiple sensors provides more information and helps better differentiating artifacts, e.g., due to eye motion or body movement. However, since the additional to EEG channels (e.g., ECG, EOG, EMG) are not always available or easy to acquire, our method relies only on EEG signals.

We propose a methodology that increases specificity in a two-stage process incorporating pattern classification. Similarly to most pattern detection methods in signal processing, the amount of data processed is reduced by first extracting candidate waveforms based on low-level detection analysis (by feature extraction), while subsequently classification is performed to maximize specificity of the overall method [3]. Specifically, the proposed method first detects candidate spikes based on a mimetic approach, and afterwards classifies the candidate spikes by embedding the data in a low dimensional space and applying supervised classification in the embedding space. The contribution of the proposed method is that (i) it is fully automated, i.e., no user interaction or manual intervention is required, (ii) it is template-free; thus it generalizes to any morphological patterns and shapes and can easily be applied for detection of other waveforms as long as some training patterns have been defined, (iii) it applies to all stages of sleep; therefore is appropriate for sleep monitoring, and (iv) it achieves high sensitivity with low false positive rate.

In the remaining part of the paper, we describe in detail the proposed methodology in Sect. 2 and report the evaluation results in Sect. 3. In Sects. 4 and 5 discussion and conclusions of this work are provided, respectively.

## Method

Interictal discharges may be morphologically divided into sharp waves, spikes, spike-wave complexes, and polyspike-wave complexes [21]. The current study focuses on EEG recordings with spikes and/or sharp waves. Spikes are transients, clearly distinguishable from background activity, with pointed peak and a duration of approximately 20–70 ms, whereas sharp waves are the same as spikes, but with a duration of 70–200 ms [21]. For simplification, we will use the term ‘spike’ both for spikes and sharp waves throughout the rest of this paper.

**Fig. 1:**
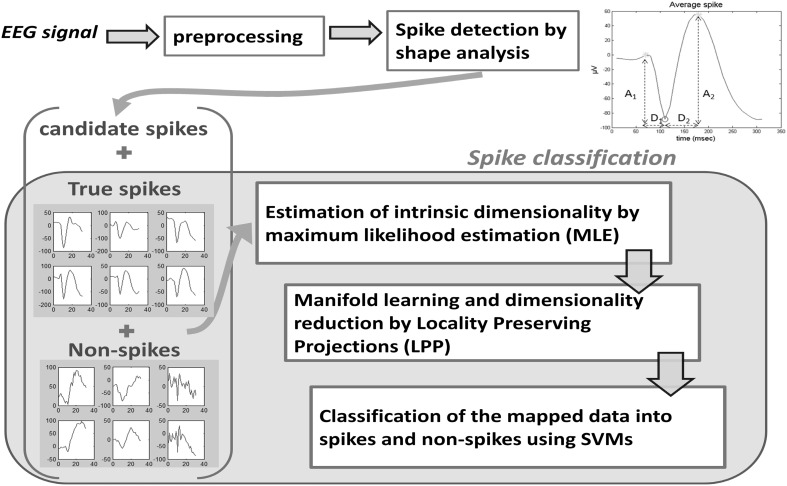
Spike detection framework. The 1st step of the method detects spike-like waveforms by extracting the two half-waves. The half-waves are defined between the negative peak (marked with a *red circle*) and the two positive peaks (marked with *green stars*) and are characterized by the amplitude difference (*A*_1_, *A*_2_) and duration (*D*_1_, *D*_2_). The 2nd step of the method classifies the detected transients into spikes and non-spikes using machine learning techniques. (Color figure online)

The proposed method first models coarsely the shape of the spike by breaking down the EEG signal around major peaks into half-waves. Thresholding of shape characteristics extracted from the half-waves, such as amplitude and duration, is applied to generate a number of candidate spike locations. Subsequently, the method classifies the candidate transients into spikes and non-spikes by learning the patterns of spikes using manifold learning, dimensionality reduction, and non-linear supervised classification. The whole pipeline of the method is illustrated in Fig. 1. The analysis involves a single time series which can be obtained by averaging the recordings of selected channels.

**Fig. 2:**
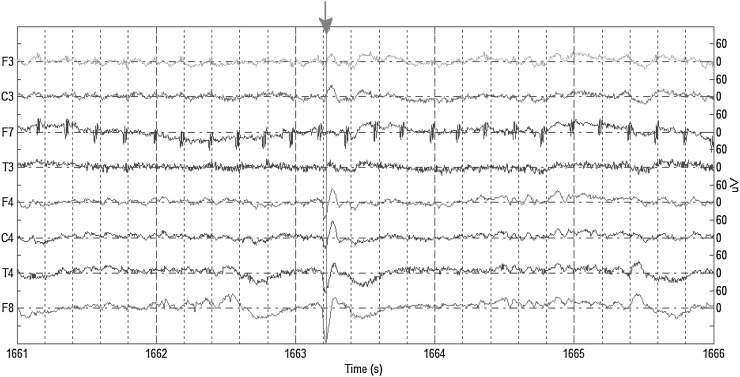
EEG recordings of selected channels showing a spike example (marked at the negative peak with a *red arrow*). (Color figure online)

Figure 2 illustrates an example of recordings of randomly selected symmetric (across the midsagittal plane) electrodes with a spike annotation. We can see that the spike does not uniformly appear in all channels but is mostly evident in the channels of the right hemisphere (in this case) and mainly in F8 electrode. The individual steps of the method are explained with more details next.

### Preprocessing

The raw EEG recordings are first downsampled (e.g., at 100 Hz) to reduce dimensionality and then a notch filter is applied with cut-off frequency at 50 Hz. Baseline correction is performed by calculating the mean signal in overlapping segments. This stepwise constant component is subsequently smoothed by using a moving average filter and subtracted from the original signal. Only the channel which clearly depicts the spikes is selected for further analysis. If more than one channels are informative, the average signal is calculated and used as input to the next step of the method performing pattern analysis. Since the same channels are also used by the expert for visual annotation, the results of the method can be easily assessed based on the temporal localization of manually and automatically detected spikes.

### Spike detection by shape analysis

First a peak detection algorithm is applied to detect the primary vertex of the spike in the form of local minima. In order to reduce the number of candidate peaks, only peaks that are at least separated by 100 ms are retained, while small peaks that may occur in close proximity to larger local peaks are ignored. Then, around each detected peak, the EEG signal is extracted within a window (starting 100 ms before the primary vertex and ending 200 ms after it) defining the spike waveform. For each waveform, the two half-waves are segmented and four time-domain parameters are calculated: the amplitude difference (*A*_1_, *A*_2_) and the duration (*D*_1_, *D*_2_) of each half-wave [15]. These parameters describe the slope around the primary vertex and are calculated as amplitude difference and time interval between the primary vertex (wave minimum) and the two closest local maxima (before and after the minimum), respectively. Figure 1 shows the three peaks: the primary vertex marked with a red circle and the two closest maxima indicated with green stars. Thresholding of the four parameters is applied to distinguish candidate spikes from other artifacts. The minimum and maximum threshold values used in this study are shown in Table 1. A maximum value on amplitude is used to discard spikes due to noise or movement. We can use amplitude thresholds because baseline correction has previously been applied causing zero-centering of the local EEG average.

**Table 1:** Threshold values for amplitude difference (*A*_1_, *A*_2_) and duration (*D*_1_, *D*_2_) of each half-wave

	Minimum	Maximum
*A*_1_	20 μV	500 μV
*A*_2_	50 μV	500 μV
*D*_1_	–	200 ms
*D*_2_	–	150 ms

It should be noted that the spike amplitudes differ between subjects, thus we relaxed the threshold constraints to make the method applicable also for “unseen” data and allow detection with high sensitivity. As a consequence, the specificity of this step becomes especially low; thus a subsequent step is required to reduce false detections using a more elaborate approach.

### Spike classification in a low dimensional space

#### Low dimensional embedding

If the raw signal (waveform around the primary vertex) is used as representation for the detected spikes, classification is deemed to fail due to the high dimensionality of the input pattern. When the number of parameters increases, the volume of the space grows so fast that the concept of similarity, distance, or nearest neighbor may not even be qualitatively meaningful, thus impeding clustering or classification. Therefore in this step of the method, the extracted candidate transients are classified either as spikes or as non-spikes by assuming that the spike and spike-like patterns reside on the same low dimensional manifold but in different regions. If this assumption holds, classification can be performed much easier after embedding the data than performed in the original high dimensional space. Thus, in this step, we first learn the low dimensional embedding using a set of spikes annotated by an expert (positive class), and a set of spike-like waves that are nonspecific sharp transients (negative class). The nonspecific sharp transients are all transients detected in the first step that were not annotated by the expert. Thus, it may be that the negative class also includes spikes that are just missed by the expert. We selected waves with spike-like patterns that satisfy the shape constraints set in the 1st step of the method as the negative class, instead of choosing random background segments, because the more similar the two classes are, the more possible it is to occupy the same manifold and thus to allow learning the separation between them.

We used the locality preserving projections (LPP) [22, 23] to embed the data in a low dimensional space. LPP is a linear approximation of the nonlinear Laplacian Eigenmap [24]. It finds a transformation matrix *A* that maps a set of points $$x_{i} \in R^{d}$$$$\left( {i = 1, \ldots ,m} \right)$$ into a set of points $$y_{i} \in R^{l}$$, $$y_{i} = A^{T} x_{i}$$, such that $$l \ll d$$. LPP is designed to preserve local structure; thus, it is likely that a nearest neighbor search in the low dimensional space will yield similar results to that in the high dimensional space. The intrinsic dimensionality (*l*) of the transients is unknown but we used the maximum likelihood estimation (MLE) method to obtain an estimated value. The MLE method gives a good estimate of the unknown parameters by maximizing the likelihood of the data we observe. It is a widely used estimation method showing essential properties with increasing number of samples, such as consistency, efficiency, and asymptotic normality.

In details, the LPP algorithm is as follows. Let *X* be the *d* × *m* matrix including the *m* waveforms. The samples constitute the nodes of a graph connected with edges having weights that depend on the samples’ distance. If *W* is the *m* × *m* weighting matrix and *D* is a diagonal matrix whose entries are column sums of *W*, the eigenvectors *α*_*k*_ and eigenvalues $\lambda_{k} \left( {k = 0, \ldots ,l - 1} \right)$ for the following generalized eigenvector problem are computed:

1 $$XLX^{T} \alpha_{k} = \lambda_{k} XDX^{T} \alpha_{k}$$

where *L* = *D* − *W* is the Laplacian matrix. The *n* × *l* transformation matrix *A* is formed by the *l* column vectors *α*_*k*_ ordered according to the corresponding eigenvalues.

#### Classification

The mapped data are subsequently introduced to an SVM classifier [25]. SVM is an extremely popular algorithm that captures complex relationships between the data points and finds an optimal boundary between the class outputs. A Gaussian radial basis function is used as kernel to perform non-linear classification. The C and γ parameters, controlling the misclassification penalty and kernel size, respectively, were defined as in [26]. Briefly, since the data are unbalanced and the sample size is rather small to produce balanced classes by subsampling the largest class, we used a weighted SVM and set the ratio of penalties for the two classes, C_1_ and C_2_, equal to the inverse ratio of the training class sizes. Thus, we avoided bias toward the class with the largest training size. We defined *γ* to be adaptive to the dimensionality *l*, using the equation $\gamma = \frac{1}{{\left( {k \cdot l \cdot log\left( l \right)} \right)^{2} }}$, where *k* is a constant determined such that the fraction of the training samples contained in the kernel is approximately 20 %.

#### Implementation details

**Fig. 3:**
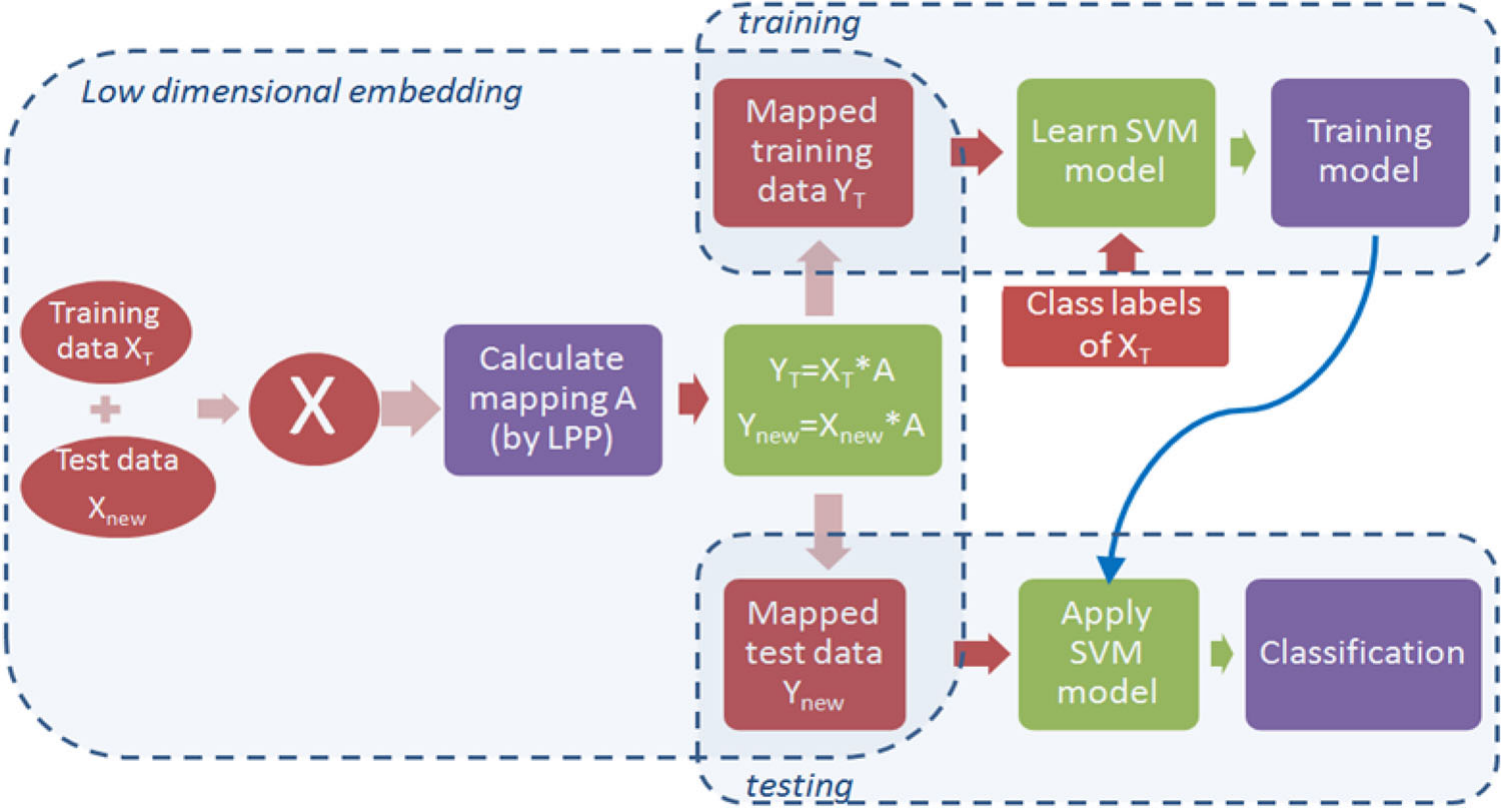
Training and testing phase of embedding and classification

The total pipeline including training and test phase is illustrated in Fig. 3. Training data *X*_T_ and test data *X*_new_ are concatenated into the matrix *X* which is used to learn the transformation matrix *A* based on LPP. In the training phase, the training data are embedded in the low dimensional space,

2 $$Y_{\text{T}} = X_{\text{T}} A$$

and then the embedded data are used in combination with the corresponding class labels to learn an SVM classification model. Similarly in the test phase, the test data are embedded in the low dimensional space,

3 $$Y_{\text{new}} = X_{\text{new}} A$$

and the embedded data are subsequently classified into spikes or non-spikes based on the learnt classification model. Since LPP supports exact out-of-sample extension, the matrix *A* could also be learnt by using the training data alone and then be applied on any new data set.

### Assessment

Assessment is performed by examining the temporal coincidence of the manually (by the expert) and automatically (by the system) detected spikes [6]. The maximum time interval between an automatically detected peak and the closest marker (detection latency), that allows a detection to be characterized as true positive (TP), is selected equal to 50 ms. A spike detected by the system with higher latency is characterized as false positive (FP), whereas the absence of a detection within the same time interval around a marker is a false negative (FN). Sensitivity is the percentage of correct detections by the system in positive events marked by the rater (TP + FN). Selectivity or precision is the percentage of correct detections by the system in positive detections (TP + FP) [6]. FP/min is the number of false positive spikes per minute of recording. A single measure of accuracy is the $F{\hbox{-}}score$ which expresses the harmonic mean of precision and sensitivity:

4 $$F{\hbox{-}}score = \frac{2TP}{2TP + FP + FN}$$

The assessment of the method refers to both steps of the method and is performed by ten-fold cross-validation in order to exploit all available data. Since the 1st step of the method is rule based (unsupervised), the cross-validation is performed only on the 2nd step including both dimensionality reduction and classification in each fold.

## Results

### EEG dataset

The EEG recordings were provided by the Epilepsy Monitoring Unit, St. Luke’s Hospital, Thessaloniki, Greece. The data used in this work were acquired during a whole-night sleep EEG of a subject with history of right lobe epilepsy of fronto-temporal origin. Nocturnal sleep was recorded using multi-channel electrodes positioned according to the extended international 10–20 system on an electrode cap with sampling frequency 500 Hz. The spikes were visually identified by an experienced neurophysiologist as transients clearly distinguished from background activity with pointed peaks. The markers were manually placed (in T4 and F8 channels) at the peak of the negative phase, but imprecise markings were later corrected by automatically shifting them to the largest negative peak within a predefined neighborhood (equal to the defined detection latency) around the original marking.

### Performance

**Fig. 4:**
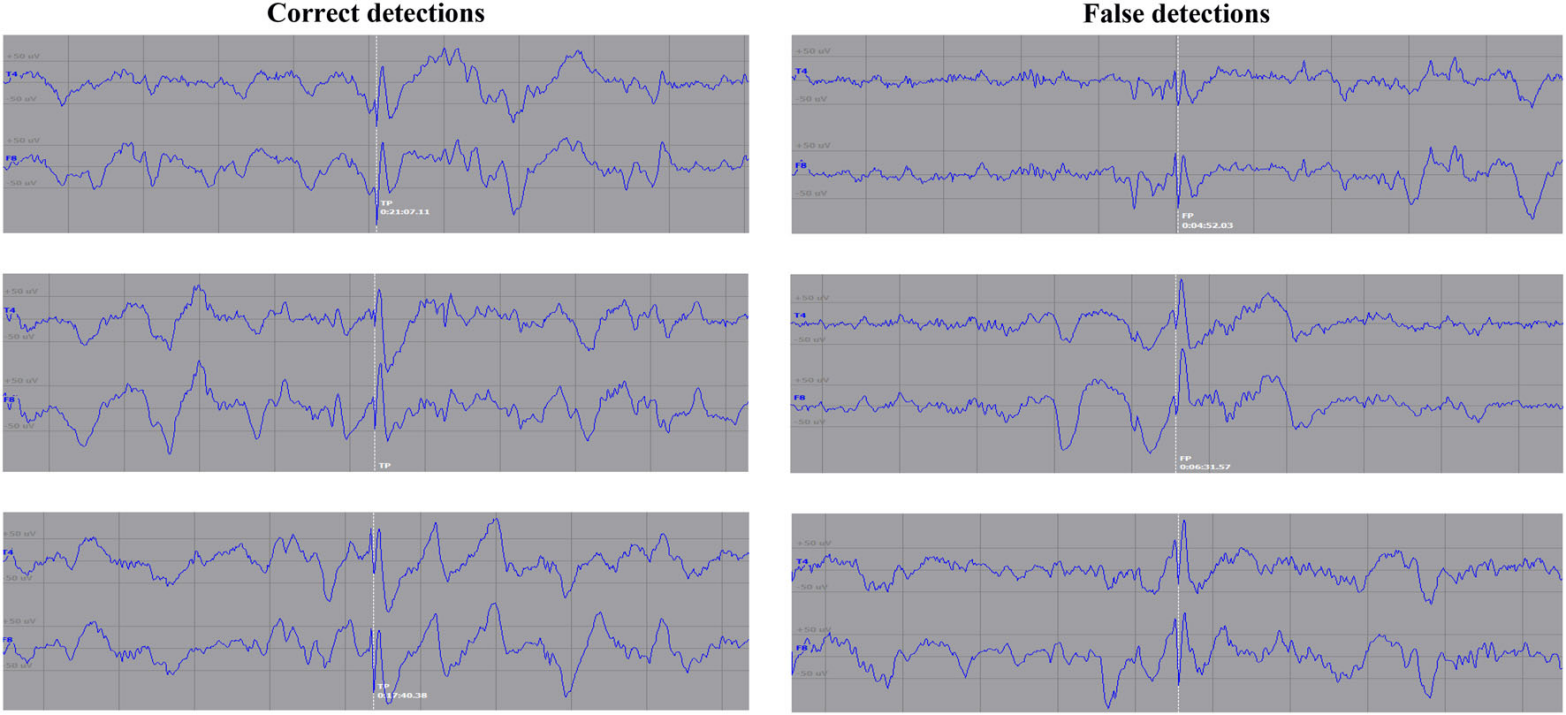
Illustration example of 3 correct (*left*) and 3 false (*right*) spike detections for the selected channels (1st channel: T4, 2nd channel: F8). The same amplitude scale (±50 μV) has been used for all plots. The spike location is approximately at the center of each plot and indicated with a *white line*

**Fig. 5:**
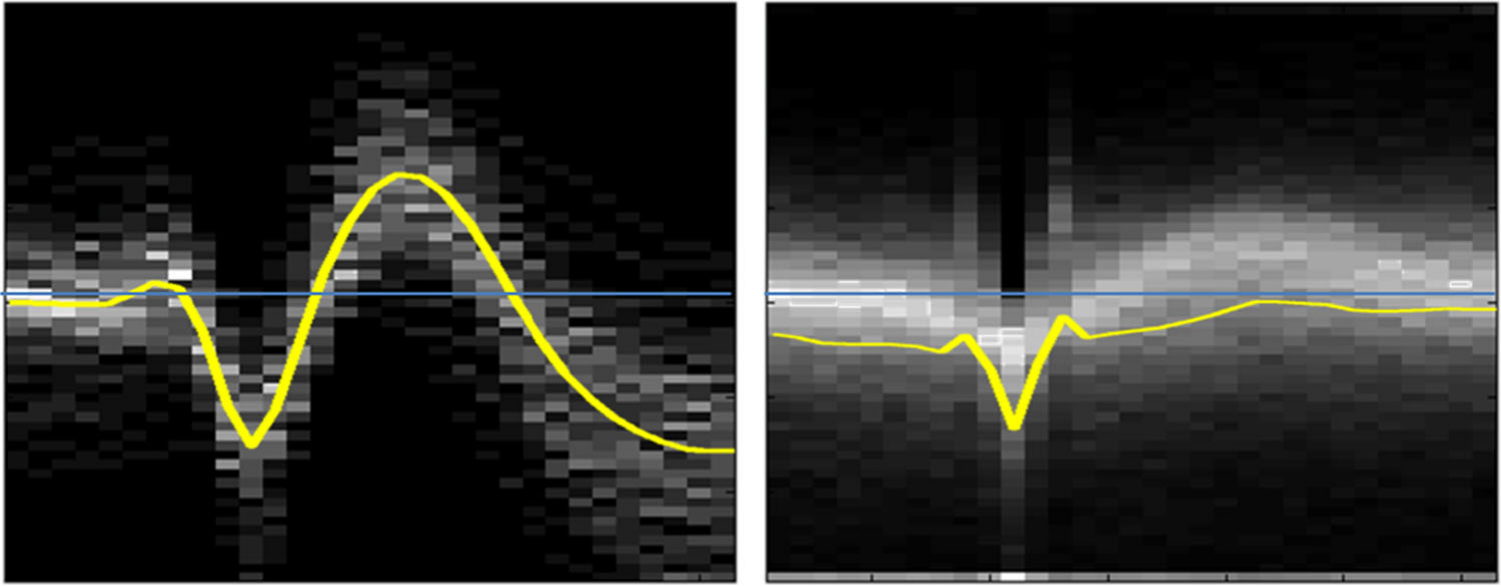
Average TP wave (*left*) and FP wave (*right*) (in *yellow*) overlaid on the corresponding probability maps obtained from all candidate transients detected in the 1st step of the method. The *blue line* indicates the zero level. The time window is 300 ms (100 ms before the primary vertex and 200 ms after it). (Color figure online)

The method was assessed on 9-h recordings including 101 marked spikes. A total number of 4708 candidate spikes (99 TP and 4609 FP) were automatically detected during the 1st step of the method. Only two spikes were missed (not detected). Examples of data containing TP and FP waveforms, identified in this step of the method, are illustrated in Fig. 4. All TP and FP transients detected in the 1st step of the method are summarized in Fig. 5 in the form of probability maps, and are also averaged to highlight shape differences between TP and FP. It is evident that, on the average, the epileptic spikes follow a more distinguishable smooth pattern than the nonspecific sharp transients. Moreover, since the FP transients are many and also exhibit large (per point) variation, the mean values do not overlap with the most frequent values.

The 2nd step of the method was assessed by ten-fold cross-validation on the data. The classification of waveforms identified 156 (out of the 4708) as spikes with 98 of them being TP and 58 being FP. Thus, the total sensitivity of the method is 0.97 (=98/101), the selectivity is 0.63, and the number of FP per minute is 0.1. The method’s performance is shown in Tables 2 and 3 and is compared against other approaches reviewed by Wilson and Emerson in [5], and by Halford [6]. Only methods for which both sensitivity and FP rate were reported are included for comparison in Table 2, whereas the rest of the methods for which both sensitivity and selectivity were reported are shown in Table 3. Studies using intracranial EEG were excluded. For some methods, more than one set of results are reported corresponding to different algorithms or parameters. If different training and testing datasets were used, this is indicated by two numbers separated by ‘/.’ Although a direct comparison is not feasible due to the different data per study, it can be seen that our method performs better than all (16) reviewed methods in Table 2, whereas it has the highest sensitivity and the 4th (out of 14) lowest selectivity among the methods reviewed in Table 3.

**Table 2:** Comparison of methods detecting epileptic activity based on FP rate

Method	No. subj.	Length (min)	No. spikes	Sensitivity	FP/min
Proposed method	1	540	101	0.97	0.1
Davey et al. [7]	1	5.3	23	0.74	0.4
Dingle et al. [8]	11	180	462	0.67	0
Witte et al. [10]	1	1	50	0.90	4.0
Senhadji et al. [11]	1?	10	982	0.86	6.8
Fischer et al. [12]	10	8	341	0.73	1.0
De Lucia et al. [13]	7	121	N/R	0.65	6.0
Gabor, Seyal [14]	5	63.8	752	0.97	1.5
Hostetler et al. [34]	5	100	1393	0.76 0.87	5.2 1.4
Webber et al. [35]	10	40	927	0.73	6.1
Feucht et al. [36].	3	90	1509	0.88	1.8
Ramabhadran et al. [18]	18	270	982	0.96	0.4
Wilson et al. [37]	50	143	1952	0.47 0.15 0.70	2.5 3.2 4.1
James et al. [38]	35/8	856/192	3096/190	0.55	0.1
Sugi et al. [39]	11	8	77	0.37	16.0
Acir et al. [40]	19/10	210/228	216/93	0.89	0.1
Argoud et al. [41]	7	N/R	6721	0.71	0.1

'?' uncertain value,* N/R* not reported

**Table 3:** Comparison of methods detecting epileptic activity based on selectivity

Method	No. subj.	Length (min)	No. spikes	Sensitivity	Selectivity
Proposed method	1	540	101	0.97	0.63
Inan and Kuntalp [15]	5/3	N/R	53/15	0.60	0.82
Indiradevi et al. [17]	22	N/R	684	0.92	0.78
Park et al. [19]	32	N/A	N/A	0.97	0.90
Ozdamar, Kalayci [20]	5	75	N/R	0.93	0.94
Goelz et al. [42]	11	278	298	0.84	0.12
Kurth et al. [43]	4	N/R	N/R	0.62	0.61
Liu et al. [44]	81	48,000	6048	0.90	0.94
Sartoretto et al. [45]	10	79	166	0.96	0.37
Latka and Was [46]	4	N/R	340	0.70	0.67
Adjouadi et al. [47]	10/21	~800	319	0.82	0.92
Adjouadi et al. [48]	9/9	~450	47/139	0.79	0.85
Exarchos et al. [49]	25	375	137	0.86	0.83
Tzallas et al. [50]	15/10	~225/150	163/111	0.89	0.83
Van Hese et al. [51]	8	130	1625	0.92	0.77

*N/A* not applicable (testing EEG dataset created in a previous study),* N/R* not reported

Furthermore, a recent method detecting interictal epileptiform discharges based on the merger of increasing and decreasing sequences and SVM classification [16] achieved average detection sensitivity ~0.96 and specificity in classification more than 0.98 in 20 min light sleep data from ten patients’ EEG recordings. Since our detection results are not intercomparable with the classification results of [16], we use for comparison the classification performance only of the 2nd step, which detected 98 (out of 99) spikes and 4551 (out of 4609) non-spikes, and thus achieved sensitivity and specificity both equal to 0.99. Detection can also be achieved through classification of all possible patterns in EEG, such as epileptiform transients (single and multiple spikes or spike-and-slow-wave complexes) and non-epileptiform transients (eye movements and artifacts), as performed in [17]. In such systems, the methods are evaluated on preselected EEG segments from each pattern category, thus the detection performance cannot directly be assessed and compared with our method in which the total recordings are used as input.

In order to assess the contribution of the selected dimensionality reduction technique, the LPP method has been replaced by other dimensionality reduction techniques [23]. The results of the best performing techniques (achieving $F{\hbox{-}}score > 0.6$) are shown sorted in Table 4. The Linear Local Tangent Space Alignment (LLTSA) [27] performs better in respect to $F{\hbox{-}}score$ but we chose LPP due to its highest sensitivity which is more important given the small value of FP/min. The neighborhood preserving embedding (NPE) [28], principal component analysis (PCA) [29], maximally collapsing metric learning (MCML) [30], stochastic proximity embedding (SPE) [31] and diffusion maps [32] also have high sensitivity with increased however FP/min. The competitive performance of the method for more than one dimensionality reduction techniques indicates the robustness of the framework to the selection of dimensionality reduction technique.

**Table 4:** Results of the best performing dimensionality reduction techniques based on *F-score*

Method	Sensitivity	FP/min	*F*-*score*
LLTSA	0.96	0.10	0.77
LPP	0.97	0.11	0.76
NPE	0.97	0.12	0.74
PCA	0.96	0.13	0.72
MCML	0.96	0.13	0.72
SPE	0.97	0.14	0.71
DiffusionMaps	0.97	0.15	0.70
LLE	0.86	0.14	0.66
MVU	0.86	0.14	0.65
NCA	0.98	0.20	0.64
LandmarkIsomap	0.93	0.19	0.64
FactorAnalysis	0.94	0.23	0.60
Isomap	0.95	0.23	0.60

**Fig. 6:**
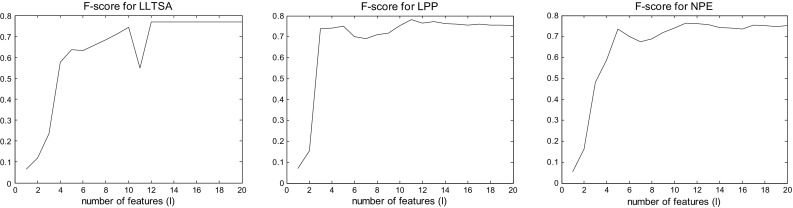
The *F-score* as a function of dimensionality for the best 3 dimensionality reduction techniques LLTSA, LPP, NPE (from *top* to *bottom*)

The original dimensionality of the waveforms used for classification was *d* = 31 (corresponding to 301 ms at 100 Hz), whereas the reduced dimensionality estimated by the MLE method was *l* = 14. The $F{\hbox{-}}score$ as a function of dimensionality is shown in Fig. 6 for the three best dimensionality reduction techniques (LLTSA, LPP, NPE). It can be seen that LPP is not very sensitive to the selection of *l* and achieves higher $F{\hbox{-}}score$ for more values of *l* than LLTSA; thus, it is preferred as technique in this application.

The method was developed in Matlab. The total computational time (including the ten-fold cross-validation scheme) in a Windows machine, Intel Core™2 Duo CPU 2.2 GHz, was approximately 4 min for the applied dataset, but depends highly on the number of candidate spikes extracted in the 1st step of the method.

## Discussion

This paper presents a system that combines a rule-based approach with machine learning for detecting interictal discharges in EEG. After the extraction of a large set of candidate spikes based on a crude shape model consisting of two half-waves, more detailed modeling of the spike waveform is performed in order to discover the non-linear structure of the data and map it to a lower dimensional space. Dimensionality reduction is performed by locality preserving projections. The main advantage of LPP is its linearity and more importantly that it is defined everywhere in ambient space rather than just on the training data points. Thus, LPP may be simply applied to any new data point to locate it in the reduced representation space. Also, LPP is derived by preserving local information; hence, it is less sensitive to outliers than PCA.

The method achieved high sensitivity with low false positive rate and outperformed the majority of the other approaches used for comparison. However, it should be noted that (i) the comparative data shown in Tables 2 and 3 (except for the proposed method) are extracted from the literature and therefore should be compared with care and (ii) the intra-subject assessment of the proposed method could affect the performance, since the recorded signals collected from different individuals can exhibit large differences, especially if there is great age difference.

The classification of events usually relies not only on the epileptic spikes themselves, but also on other contextual information such as spatial information (same event in other channels) and temporal information (time shift of the event). We did not make use of spatial and/or temporal context in the system. We also did not use contextual information on the surrounding background EEG. The additional spatial and temporal cues do not seem to be very important in this intra-subject analysis of single spikes where validation is performed on the channels used during visual annotation.

The development of a single-channel method is preferred because it does not necessitate multi-channel recordings. If we want to detect spikes on new patients with no prior information (unknown seizure origin and unavailable individualized annotations), we can apply the method on each channel independently and then apply a basic spatiotemporal fusion rule. Such a rule could impose spatial and temporal constraints on the per-channel detections in order to differentiate between TP and FP. As an example, a spike should appear in at least 2 neighboring channels within 20 ms distance between the detections. Such rules are common in EEG waveforms detections [18, 33]; however, they require a larger number of annotated recordings (than currently available to us) for testing their generalization ability.

The aim of this study was to achieve high sensitivity minimizing missed events, even at the expense of reduced specificity, because the detected events can later be checked by a neurophysiologist. Our aim was the reduction of the time needed to analyze long sleep recordings through the use of an automated tool estimating interictal spike frequency. Such a tool might be especially useful in the analysis of inoperable epilepsy, such as childhood absence epilepsy, since the relative reduction in spike frequency indicates effective treatment.

## Conclusion

In this work, we introduced a machine learning approach for personalized detection of focal EEG abnormalities, such as spikes and sharp waves, necessary for the automated assessment of the clinical implications of a recording. Despite not directly comparable, the presented method has higher sensitivity (=97 %) and smaller FP rate (=0.1 min^−1^) than most approaches proposed in the literature, thus constitutes a useful tool for automated assessment of interictal discharges in sleep EEG. Moreover, it is fully automated, template-free and can be easily extended to the detection of other waveforms. The method has not been applied in long-term (24 h) EEG recordings where physiological artifacts (from speaking, eating, etc.) disturb the signal and make interpretation much more difficult. Also, further evaluation on data from multiple individuals is required to assess the inter-subject performance.
